# Predictors of the one-year-change in depressiveness in informal caregivers of community-dwelling people with dementia

**DOI:** 10.1186/s12888-021-03164-8

**Published:** 2021-04-03

**Authors:** Lara Kürten, Nikolas Dietzel, Peter L. Kolominsky-Rabas, Elmar Graessel

**Affiliations:** 1 Interdisciplinary Center for Health Technology Assessment (HTA) and Public Health (IZPH), Friedrich-Alexander University Erlangen-Nürnberg (FAU), Erlangen, Germany; 2 Center for Health Services Research in Medicine, Department of Psychiatry and Psychotherapy, University Hospital Erlangen, Friedrich-Alexander University Erlangen-Nürnberg (FAU), Erlangen, Germany

**Keywords:** Dementia, Informal caregivers, Depressiveness, Predictors, WHO-5, Longitudinal, Supervision time

## Abstract

**Background:**

The care of people with dementia is usually carried out by their family members, which can cause objective und subjective burden and raise their risk of depressiveness. Thus, the aim of this study is to identify predictors of the change in depressiveness of informal caregivers over 1 year in order to be able to derive hypotheses for interventions that promise success.

**Methods:**

The Bavarian Dementia Survey (BayDem) is a multi-center, longitudinal study conducted at three different sites in Bavaria, Germany. Participants were people with dementia and their informal caregivers. Data was collected at baseline and after 12 months by standardized face-to-face interviews in cooperation with local players. The informal caregivers’ depressiveness was assessed with the WHO-5. Data was also collected on the people with dementia’s cognition (MMSE), behavioral symptoms (NPI) and comorbidities (Charlson Comorbidity Index) as well as caregivers’ social inclusion (LSNS), time spent on care and care contribution (RUD). For statistical analysis, a multiple regression model was used.

**Results:**

The data of 166 people with dementia and their informal caregivers was analyzed. Of the latter, 46% were categorized as “likely depressed”. The change in depressiveness over a year was significantly predicted by baseline depressiveness as well as an increase in the time informal caregivers spent supervising the person with dementia.

**Conclusions:**

Informal caregivers of people with dementia are at high risk of depression. The time spent supervising the person with dementia has a significant impact on increasing depressiveness. This highlights the importance of support services to provide the informal caregiver with relief and possibly reduce depressiveness.

## Background

One third of informal caregivers of people with Alzheimer’s dementia suffer from depression – a percentage greater than the prevalence of depression in the general population or even among caregivers to patients of other psychiatric or physical illnesses [1, 2]. In addition to the harmful effects for those affected, depression also leads to high direct and indirect costs in Germany every year [3]. In 2018, depressive episodes were the third most frequently reported cause of absences from work [4]. Informal caregivers‘depressiveness in particular has been associated with, among other things, higher health care costs [5], suicidal ideation [6], more frequent conversion to nursing homes of the person with dementia (PWD) [7, 8], and more cardiovascular diseases of the informal caregiver [9].

Seeing as dementia is on the rise both in Germany and worldwide [10, 11] and most PWD are cared for at home by their relatives rather than moving to a nursing home [12, 13], there is a pressing need to determine which factors have a central influence on the depressiveness of informal caregivers to be able to derive interventions. Multiple systematic reviews have been written on this topic. Common predictors and correlates of caregiver depression include behavioral and psychological symptoms, female gender of caregivers, being the spouse of the PWD, low social support, competence and coping strategies of the caregiver [1, 14–16]. Other studies have found correlations between depression and dementia severity [17, 18], lower caregiver education [19, 20], or ADL dependence [21]. However, two facts stand out when reviewing previous literature on this topic: for one, most studies feature a cross-sectional design [14, 15], which makes the direction of causation unclear. And for another, few studies are based on a theoretical framework, seeming to choose their sets of analyzed predictors more due to the convenience of available data from existing studies (for an exception of a study embedded in a theoretical model, see for example Piercy et al. [19]). That is why the current study aimed to a) employ a longitudinal design in its prediction of change in depressiveness in addition to the cross-sectional view, and b) base the selection of predictors on a theoretical model. The current study refers to data from a dementia register, which collected a wide range of variables over multiple points in time. To select potential influencing factors on the depressiveness of informal caregivers out of this plethora of variables on a theoretical basis, the authors used the stress process model by Pearlin et al. [22]. This model examines how stress of informal caregivers arises in the care of PWD and considers different groups of predictors, mediators and outcomes (including depression).

The research question addressed here is: What factors influence the change in depressiveness of informal caregivers of a community-dwelling PWD over the course of 1 year?

## Methods

### Study design and study population

The Bavarian Dementia Survey (BayDem) is a multi-center longitudinal study conducted in three regions of Bavaria – Dachau, Erlangen and Kronach [23]. The latter represent different demographic and socioeconomic areas with different population trends. By including cities and rural districts, both urban and rural areas were represented. The division of the places of residence of all participants into urban and rural was carried out by the *Bundesinstitut für Bau, Stadt und Raumforschung* (Federal Institute for Research on Building, Urban Affairs and Spacial Development) [23]. Participants were both the PWD (defined according to the International Statistical Classification of Diseases and Related Health Problems, 10th Revision – ICD-10, F00-F03), who were cared for by their informal caregivers at home, and the informal caregivers themselves. Informal caregivers were defined as the main support persons living with or close to the PWD, such as partners, children, children-in-law, or close friends, who did not receive payment for their care of the PWD. Inclusion criteria for the PWD were a dementia diagnosis according to ICD-10 and living in the home environment in one of the three project regions. PWD were excluded if they had a severe psychiatric diagnosis other than dementia (such as schizophrenia, depression, or addiction), lived in a nursing home, or had no informal caregiver.

### Recruitment and follow-up

In order to take into account the different access routes of PWD, participants were recruited through a variety of institutions (counseling centers, doctors and therapists in private practice, medical care centers, memory clinics, nursing services, volunteer services and hospitals). For this purpose, the local players were identified and integrated into the project. The data collection was carried out by trained interviewers in the form of standardized personal interviews with both the PWD and their caregiver in the home environment. The data were collected at study entry (t0) and after 12 months (t12).

### Theoretical background

Table 1 lists the different groups of variables in Pearlin et al.’s stress process model [22] and which of these constructs were measured in the dementia registry at hand as well as their operationalization within the data set.

**Table 1:** Overlap of BayDem variables and Pearlin’s stress process model

Category	Variable	Measures in BayDem
Background and Context	SES Characteristics *Age, sex, education*	sociodemographic information
	Caregiving History *Relation to PWD* *Comorbidities of PWD*	sociodemographic information Charlson Comorbidity Index
	Family and Network Composition *Social network of informal caregiver*	LSNS
	(Program Availability)	n.m.
Primary Stressors	Objective Indicators Cognitive Status C*ognition* Problematic Behavior *Behavioral symptoms in dementia* *Necessary surveillance* ADL, iADL Dependencies *Time spent on care*	MMSE NPI Supervision care time (RUD) ADL, iADL care time (RUD)
	(Subjective Indicators)	n.m.
(Secondary Role Strains)		
(Secondary Intrapsychic Strains)		
Mediators	(Coping)	n.m.
	Social Support *Contribution to care*	RUD
Outcomes	Depression *Depressiveness* (Anxiety) (Irascibility) (Cognitive Disturbance) (Physical Health) (Yielding of Role)	WHO-5 n.m. n.m. n.m. n.m. n.m.

Source: Pearlin et al. [22]

*n.m.* not measured, *PWD* person/people with dementia, *ADL* activities of daily living, *iADL* instrumental activities of daily living. *Italics*: operationalization in BayDem. Constructs in brackets were not encompassed in the BayDem data set. Only depression was considered as an outcome as it is the main focus of the present study

### Measures

Sociodemographic data of the PWD and informal caregivers were collected. The risk for 1-year mortality of PWD and informal caregivers based on physical comorbidity was assessed with the Charlson Comorbidity Index [24]. The higher the score, the more (and the more severe) comorbidities were listed by the participants, with a maximum score of 36. The Lubben Social Network Scale (LSNS) [25] was used to measure the informal caregiver’s social inclusion in a network. The higher the score on a range of 0 to 60, the larger the social network. Cognitive function of the PWD was assessed using the Mini Mental State Examination (MMSE) [26], resulting in scores between 0 and 30. Lower scores indicate worse cognitive functioning. Psychological and behavioral symptoms of the PWD were recorded with the Neuropsychiatric Inventory (NPI) [27] on a range of 0 to 144, with higher scores indicating more behavioral symptoms.

The time spent on care by the informal caregiver was determined using the Resource Utilization in Dementia (RUD) questionnaire [28]. The RUD asks for the time spent on care on a normal day of care and the number of days within the last 30 days which were spent caring for the PWD. The two values were multiplied by each other and divided by 30 to calculate the actual average care time per day during the last month in a way that is comparable between participants. Care time is surveyed for three areas: assistance with activities of daily living (ADL), assistance with instrumental activities of daily living (iADL), and supervision of the PWD. Another item records the contribution of care the interviewed informal caregiver provides to the PWD in relation to other informal carers: “Considering all caregivers, how large is your contribution?”. It is explicitly noted that outpatient care, 24-h-care, household helps, or other institutional help are not taken into account, only other carers from the personal environment. Thus, the item could be considered as an indirect assessment of the informal caregiver’s social support in that it measures the help received by family and friends in caring for the PWD. The informal caregiver’s care contribution is assessed on a five-point scale of 1 = *1–20%,* 2 = *21–40%*, 3 = *41–60%*, 4 = *61–80%,* and 5 = *81–100%*.

The subjective burden on informal caregivers was determined using the 10-item shortform of the Burden Scale for Family Caregivers (BSFC-s) [29], with a range of 0 to 30 and higher scores indicating higher burden. The above-mentioned measures are internationally used and validated for use with PWD [28, 30–34].

The central instrument of this work is the WHO-Five Well-being Index (WHO-5), which was derived from the WHO-10 [35] and is used as a valid screening tool for depression [36]. Higher scores on this scale indicate low depressiveness of the informal caregiver and vice versa. Depressiveness is therefore understood as a continuum in this article. The WHO-5 consists of five items (e.g. “I have felt cheerful and within good spirits”) relating to the past 2 weeks, with a response format from 0 = *at no time,* 1 = *some of the time*, 2 = *less than half of the time*, 3 = *more than half of the time*, 4 = *most of the time*, to 5 = *all of the time*. Thus, values from 0 to 25 can be obtained. As suggested in the systematic review by Topp et al. [36], the values were multiplied by 4 to achieve a range of values from 0 to 100. This is a common practice used to increase comparability with scales measuring health-related quality of life where this percentage format is customary, and was used here to better be able to compare the results with those of other studies using the WHO-5. In addition, dichotomization was performed at the suggested cut-off of 50 into “likely depressed” (0 - 50) and “unlikely depressed” (51–100). In previous studies, this cut-off showed an acceptable sensitivity (0.87 on average) and specificity (0.76 on average) when compared to e.g. clinical structured interviews when screening for depression [36].

Apart from unchangeable demographic data, all measures were collected at both t0 and t12.

### Statistical analysis

Those dyads of PWD and informal caregiver were selected where the informal caregiver had answered the WHO-5 at both t0 and t12. ANOVAs, Welch-tests (in case of heterogeneous variances), and Mann-Whitney-U tests (for ordinal scaled variables) were used to check if missing values in depressiveness at t12 were missing at random by comparing for relevant study variables between those dyads where the informal caregiver had given information about their depressiveness only at t0 vs. both at t0 and t12. In accordance with the S3 Dementia Guideline [37] as well as the suggestions of the National Institute for Health and Care Excellence (NICE) of the UK [38], those dyads were also excluded in which the MMSE value of the PWD was > 26, as the presence of dementia is unlikely. For all sub-analyses with care time, those dyads were also excluded whose sum of ADL, iADL and supervision per day was more than 24 h. Relevant characteristics of this group at t0 are descriptively presented, as well as the frequency of the dichotomized groups “likely depressed” and “unlikely depressed” and their change from t0 to t12. The one-year period was chosen in order to reduce seasonal influences. The change in the depressiveness score (WHO-5 at t12 minus WHO-5 at t0) was also calculated over a one-year period.

A multiple regression model was developed to identify factors influencing this change in the depressiveness score. Variables from the BayDem register were considered as potential predictors in the model if they are compatible with the stress process model according to Pearlin et al. [22] (see Table 1). Only those variables were included in the model that showed a significant bivariate relationship with the target variable (change in depressiveness). For modifiable predictors (e.g. cognition), the change in the variable over 1 year was additionally calculated and tested for significant correlation with the target variable. An adjustment to the initial score was made, i.e. the initial score of depressiveness at t0 was included in the statistical model as a predictor for the change in depressiveness over time, as well as the initial score of significantly correlating change scores of modifiable variables. Age and gender of PWD and informal caregiver were added as control variables. Multicollinearity of the variables was checked using the VIF coefficient and the intercorrelation of the variables. The variable with the higher VIF coefficient was excluded. The data were analyzed and evaluated using SPSS Software, Version 24. A *p*-value < .05 was considered statistically significant.

## Results

### Sample description

Of the 364 PWD and 339 informal caregivers which participated in BayDem at baseline, 166 dyads met the above criteria as outlined under ‘statistical analysis’. One hundred eigthy-nine informal caregivers provided information on their depressiveness at t0 and t12. Compared to the 141 dyads where informal caregivers had given information on their depressiveness at t0 only, these 189 dyads consisted of significantly younger PWD (F(1, 329) = 6.47, *p* = .011), with higher levels of cognition (Welch’s F(1, 255.80) = 10.89, *p* = .001) and less behavioral symptoms (F(1, 318) = 4.26, *p* = .040), as well as less comorbidities in the informal caregivers (Welch’s F(1, 168.77) = 4.67, *p* = .032). No difference was observed on the initial level of depressiveness (F(1, 329) = 1.61, *p* = .205), indicating the missing values to be missing at random. Of the 189 dyads, 23 dyads were excluded because the PWD had an MMSE > 26. In addition, for all subanalyses with care time, 6 dyads were excluded because their sum of ADL, iADL and supervision time per day exceeded 24 h.

Most of the PWD had the diagnosis ‘unspecified dementia’ (F03, 30%), followed by ‘dementia in Alzheimer disease with late onset’ (F00.1, 18%) and ‘dementia in Alzheimer disease, atypical or mixed type’ (F00.2, 11%). For 14% of participants, information about the diagnosis was not yet available at baseline for various reasons. All other diagnosis subcategories had group sizes smaller than 5%.

Tables 2 and 3 show the characteristics of the sample at t0. Two-thirds of the informal caregivers were female, while the proportion of women among the PWD was more balanced at 55%. The informal caregivers were distinctly younger (M = 61.8 years, SD = 12.9) compared to the PWD (M = 78.8 years, SD = 8.8). In about half of the cases (*n* = 79), the informal caregivers were the partners of the PWD. In the remaining 86 cases, the majority (*n* = 67) were adult (in-law) children. Since few informal caregivers (*n* = 19) indicated a different relationship, the variable “relation to the PWD” was dichotomized into partner and non-partner. Partners were significantly older than non-partners (*r* = .70, *p* < .001) and more often lived in the same household as the PWD (*r* = .73, *p* < .001). With a mean value of 11.5 (SD = 7.9), the informal caregivers were on average moderately burdened by the caregiving (for norm values see [30]). Measured by the mean value of 17.9 points for the MMSE (SD = 5.9), the PWD were on average moderately cognitively impaired [37].

**Table 2:** Characteristics of PWD and the care situation at baseline and correlations with depressiveness

	t0		r (p)		
	N	n (%) / M (SD)	Depressiveness t0	Depressiveness t12	Depressiveness t12-t0
**PWD**					
Age ^c^	166	78.8 (8.8)	−.11 (.151)	−.02 (.838)	.10 (.221)
Female ^c^	166	91 (54.8%)	.07 (.392)	**.22 (.004)**	**.18 (.023)**
Cognition (MMSE) ^a,c^	147	17.9 (5.9)	.07 (.408)	.09 (.309)	.03 (.756)
∆ Cognition (MMSE) ^a,b^	118	−1.9 (5.4)	−.07 (.483)	**.18 (.049)**	**.28 (.002)**
Comorbidities (Charlson Comorbidity Index) ^a^	148	1.9 (2.1)	**−.26 (.001)**	−.10 (.213)	.15 (.071)
∆ Comorbidities (Charlson Comorbidity Index) ^a^	137	−0.4 (1.7)	.05 (.589)	−.03 (.744)	−.08 (.374)
**Care situation**					
Behavioral and psychological symptoms (NPI) ^a^	161	23.4 (20.8)	**−.37 (< .001)**	**−.31 (< .001)**	.04 (.610)
∆ Behavioral and psychological symptoms (NPI) ^a^	146	−2.8 (17.9)	.09 (.296)	.10 (.249)	.02 (.793)
Average care time in hours per day (RUD)					
Help with ADL ^a^	152	1.1 (1.6)	−.14 (.102)	−.10 (.233)	.03 (.775)
∆ Help with ADL ^a^	138	0.1 (1.3)	.01 (.893)	−.10 (.221)	−.13 (.128)
Help with iADL ^a^	152	2.1 (2.1)	−.08 (.330)	−.08 (.361)	−.00 (.966)
∆ Help with iADL ^a^	136	0.0 (2.0)	−.01 (.907)	−.04 (.666)	−.03 (.703)
Supervision ^a,c^	149	3.3 (5.0)	**−.29 (.001)**	**−.34 (< .001)**	−.09 (.285)
∆ Supervision ^a,b^	134	0.5 (4.1)	.11 (.190)	−.08 (.382)	**−.20 (.024)**

*PWD* person/people with dementia, *ADL* activities of daily living, *iADL* instrumental activities of daily living. ∆ = change t12-t0. ^a^ = considered for the regression model according to Pearlin‘s stress process model. ^b^ variable congruent with Pearlin’s stress process model included in the regression model as predictor due to significant correlation. ^c^ variable added to the regression model as predictor due to methodological considerations. Correlation coefficients (r) used: Pearson for interval scale variables, Spearman for ordinal scale variables. Bold = significant on the level *p* < .05

**Table 3:** Characteristics of informal caregivers at baseline and correlations with depressiveness

	t0		r (p)		
	N	n (%) / M (SD)	Depressiveness t0	Depressiveness t12	Depressiveness t12-t0
**Informal caregivers**					
Age ^a,b,c^	165	61.8 (12.9)	.08 (.331)	**−**.10 (.201)	**−.19 (.017)**
Female ^a c^	165	118 (71.5%)	**−.20 (.010)**	**−.23 (.004)**	−.05 (.567)
Relation to PWD (partner) ^a,b^	165		.05 (.513)	**−**.14 (.067)	**−.21 (.007)**
Child, relative, other		86 (52.1%)			
Partner		79 (47.9%)			
Same household as PWD	165	105 (63.6%)	−.03 (.747)	.**16 (.042)**	−.15 (.058)
Highest school degree ^a^	164		.05 (.554)	**.16 (.037)**	.13 (.098)
No school degree		1 (0.6%)			
Volksschule (primary school)		34 (20.7%)			
Hauptschule (secondary school)		48 (29.3%)			
Mittlere Reife (secondary school certificate)		48 (29.3%)			
Fachhochschulreife (advanced technical college certificate)		11 (6.7%)			
Abitur (high school certificate)		22 (13.4%)			
Place of residence (rural)	166		**−.21 (.008)**	−.15 (.050)	.04 (.595)
Urban		112 (67.5%)			
Rural		54 (32.5%)			
Social inclusion (LSNS) ^a^	118	30.5 (9.9)	**.22 (.017)**	**.29 (.001)**	.09 (.316)
∆ Social inclusion (LSNS) ^a^	114	−0.6 (8.3)	−.03 (.782)	.03 (.731)	.06 (.532)
Care contribution (RUD) ^a^	138	4.3 (1.11)	**−.29 (.001)**	**−.19 (.024)**	.06 (.486)
∆ Care contribution (RUD) ^a^	121	0.2 (1.0)	.00 (.996)	.03 (.768)	.04 (.688)
Caregiver burden (BSFC-s)	162	11.5 (7.9)	**−.57 (< .001)**	**−.46 (< .001)**	.07 (.388)
Comorbidities (Charlson Comorbidity Index)	124	0.6 (1.0)	−.10 (.274)	−.12 (.203)	−.03 (.774)
Depressiveness (WHO-5) ^c^	166	51.2 (23.6)	.	**.55 (< .001)**	**−.41 (< .001)**

*PWD* person/people with dementia. ∆ = change t12-t0. ^a^ = considered for the regression model according to Pearlin‘s stress process model. ^b^ variable congruent with Pearlin’s stress process model included in the regression model as predictor due to significant correlation. ^c^ variable added to the regression model as predictor due to methodological considerations. Correlation coefficients (r) used: Pearson for interval scale variables, Spearman for ordinal scale variables. Bold = significant on the level *p* < .05

Compared to a German norm sample [39] of similar age (M_WHO-5_ = 66.8, SD _WHO-5_ = 20.5), the informal caregivers of the present sample (M_WHO-5_ = 51.2, SD _WHO-5_ = 23.6) were above average depressed. This difference is statistically significant, t(165) = − 8.49, *p* < .001. In relation to the cut-off of 50, 46% of the informal caregivers fall into the range where depression is likely (see Table 4). The arithmetic mean of the depressiveness score according to WHO-5 barely varies in the sample within 12 months (M_t12_ = 50.9; SD_t12_ = 25.6). However, 18% change from the likely depressed group to the unlikely depressed group and 13% vice versa. The change scores of depressiveness from t0 to t12 show a wide range (from − 68 to + 64, SD = 23.4) with approximately normal distribution. Thus, even though the mean of depressiveness at t0 lies close to the cutoff, it seems unlikely that group changes were merely caused by minor fluctuations of the score.

**Table 4:** Means of informal caregiver depressiveness and dichotome groups according to cut-off ≤50

	t0		(to) t12	
**N**	166		166	
	**M**	**SD**	**M**	**SD**
Depressiveness (WHO-5)	51.2	23.6	50.9	25.6
	**n**	**%**	**n**	**%**
**likely depressed**	77	46.4%	70	42.2%
**change from t0**				
stayed likely depressed			48	28.9%
turned likely depressed			22	13.3%
stayed unlikely depressed			67	40.4%
turned unlikely depressed			29	17.5%

At baseline, the depressiveness of informal caregivers was significantly higher when the PWD had more comorbidities and more psychological and behavioral symptoms, and also when the informal caregiver spent more time supervising the PWD, was female, resided in a rural area, had low social inclusion, high personal contribution to care, and high caregiver burden (see Tables 2 and 3). An increase in the depressiveness of informal caregivers over 12 months had a significant bivariate association with the gender of PWD (more common in caregivers of male PWD), their declining cognition, an increase in supervision time, higher age of informal caregivers at baseline, being the partner of the PWD, and low baseline depressiveness (Tables 2 and 3).

### Multivariate factors influencing the change in depressiveness

In Tables 2 and 3, those variables that were considered for the regression model due to Pearlin’s stress process model are marked with an ^a^. Of these, the variables that correlated significantly with the change in depressiveness scores are marked with a ^b^. These variables were included in the regression model as potential predictors, and additionally, in the case of the change variables, the initial value of the respective variable for adjustment as well as gender and age of PWD and informal caregivers as basic demographic control variables and baseline depressiveness for adjustment. These variables added due to methodological considerations are marked with a ^c^. The variable “relation to the PWD” was excluded due to multicollinearity (VIF = 7.79). As mentioned above, older informal caregivers were significantly more often the partner of the PWD.

Due to listwise exclusion of cases, the regression featured a final sample size of 98. As Table 5 shows, the multiple regression model used to predict the change in depressiveness over 1 year was significant, F (8, 89) = 4.20, *p* = < .001, *R*^*2*^ = 0.23. In addition to low baseline depressiveness, an increase in supervision time proved to be a significant predictor of worsening depressiveness. High supervision time at t0 lay at the threshold of significance with *p* = .058.

**Table 5:** Predictors of the change in informal caregiver depressiveness from t0 to t12

	Kor. R^**2**^	SE	F (8,89)	p	n
	.229	19.70	4.20	< .000	98
**Variable**	**b**	**SE**	**Β**	**p**	**VIF**
Constant	2.62	27.70		.925	
**Depressiveness t0 (WHO-5)**	− 0.35	0.09	**−.35**	< .001	1.11
Age PWD	−0.01	0.28	−.00	.974	1.21
Gender PWD (female)	9.60	5.82	.21	.103	2.13
Age informal caregiver	0.09	0.19	.05	.619	1.49
Gender informal caregiver (female)	−2.06	5.96	−.04	.730	1.56
Cognition t0 (MMSE)	0.16	0.38	.04	.682	1.33
∆ Cognition (MMSE)	0.48	0.42	.12	.256	1.42
Supervision t0 (RUD)	−1.12	0.58	−.25	.058	2.12
** ∆ Supervision (RUD)**	−1.46	0.56	**−.29**	.010	1.55

*PWD* person/people with dementia. ∆ = change t12-t0. Bold = significant on the level *p* < .05

## Discussion

The present study is a multicenter investigation in three regions in the Free State of Bavaria. The large number of variables included as well as the longitudinal design allowed for the investigation of influencing factors of the change in depressiveness of informal caregivers of PWD. The data of 166 informal caregivers and their PWD were analysed, showing higher depressiveness than in a German norm sample of similar age, with 46% screening as depressed. Interestingly, an increase in supervision time over 1 year significantly predicted an increase in depressiveness over the same timespan, even when controlling for age, gender, baseline depressiveness and cognition.

As has been shown in other studies [1, 2], informal caregivers of PWD represent a risk group for depression. Almost half the group of participating informal caregivers in this study seemed likely to have developed clinically relevant symptoms of depression and should be advised to seek further diagnosis. What is it that puts informal caregivers of PWD more at risk of negative mental outcomes than the general population or even caregivers of other illnesses? The fact that caregiving overall negatively affects psychological health is a widely-known phenomenon and attributed in part to the fact that caregiving “often restricts the personal life, social life, and employment of the caregiver” ([40], p.250) and produces uncertainty due to the unforeseeable evolvement of the care receiver’s symptoms. Now, with dementia in particular, that strain is heightened further due to the presence of behavioral and psychological symptoms of the PWD, such as aggression, paranoia, or sleep disturbances. Behavioral and psychological symptoms in dementia have been linked to poor mental health of informal caregivers of PWD [14, 41].

The high prevalence of depressive symptoms in informal caregivers of PWD highlights the importance to act and find modifiable factors to reduce mental distress, especially in light of the high resulting cost for society as a whole (e.g. higher health care cost [5]) and the affected people themselves (e.g. higher suicidal ideation [6]). Cross-sectional connections to depressiveness in this study are similar to those found in previous research (e.g. behavioral and psychological symptoms [14], social support [17, 42], or female gender of PWD [1]). This holds particularly true for the close connection between depressiveness and caregiver burden. In their meta-analysis of 2019, Del-Pino-Casado et al. found correlations of around *r* = .5 between depression and subjective caregiver burden among caregivers in general, with even higher values among caregivers of PWD [43]. These findings are very similar to the correlation coefficient of *r* = .57 observed in this study.

However, a look at the longitudinal data seems interesting. If one controls for age and gender of PWD and informal caregivers as well as the baseline score of depressiveness, cognition or its decrease no longer show a significant association with the change in depressiveness, but supervision time (marginal) as well as its increase do. This might indicate that it is not the disease symptoms per se that are responsible for the poor mental health of informal caregivers, but rather the resulting need for supervision of the PWD. Also, caregivers higher in age were usually the partner of the PWD and more often lived in the same household. Yet similar to cognition, age of the caregiver lost its significant association with change in depressiveness when in a model with supervision time. In that vein, it might not be the high age of the caregiver or their subsequent state as a partner to the PWD living in the same household which might increase mental distress by having to provide a deeper level of caregiving [1, 41] or due to the difficult adjustment to a changed relationship [44], as previous studies have suggested, but rather the strain of constant vigilance emerging from rising supervision time. This might also be one of the reasons why informal caregivers of PWD are more depressed than caregivers of other groups, as supervision time is higher in the care of PWD than of people without dementia and has been shown to rise with increasing dementia severity [45–48].

Even though with an R^2^ of 0.23, 77% of the variance in change in depressiveness is still left unaccounted for in the regression model, the coefficient of determination can be interpreted as almost reaching a large effect (*R*^*2*^ = 0.26) according to Cohen [49]. Change in supervision time was one of only two predictors to reach significance in this model. But why might supervision of the PWD be so burdensome to the caregiver? Supervision is by far the most time-consuming part of informal care when compared to helping with ADL and iADL [47, 48, 50]. Informal caregivers have to prevent dangerous events, like falls or accidents with fire, manage behavioral symptoms, such as wandering or aggression, and even deal with potentially embarrassing behaviors, such as urinating on the floor [51]. Studies show that the informal caregivers often feel the need to constantly supervise, just in case something happens [51, 52], and it might be that strain of constantly fearing for something dangerous or embarrassing to happen to their care receiver which causes stress to the informal caregiver. Stress is the basis of Pearlin’s stress process model, leading eventually to such negative outcomes as depression [22]. Perhaps an important mediating factor here might be anxiety of the informal caregiver, which has been linked to behavioral disturbance in dementia [15, 53], which in turn correlates with supervision time [51]. Anxiety has been shown to often occur alongside depression [54, 55]. Anxiety was unfortunately not a part of this data set and is rarely the focus of research concerning informal caregivers [15], but could help illuminate the relationship between supervision time and depression. Further research is needed to examine whether behavioral and psychological symptoms in dementia and the consequent anxiety over the PWD entering dangerous or embarrassing situations perhaps increase both supervision time and caregiver depressiveness.

Furthermore, the current study used Pearlin’s stress process model [22] as theoretical framework for the selection of variables. Unfortunately though, two categories of predictors – secondary role strains and secondary intrapsychic strains – could not be fitted to variables assessed within the BayDem data set. Future research might benefit from investigating the impact on caregiver depression under account of *all* categories of Pearlin’s model in a longitudinal study design.

### Strengths and limitations

As there is no population register for informal caregivers of PWD that could be used for recruitment, the study population is not representative for all informal caregivers in Bavaria. However, recruitment of participants took place through a variety of institutions, such as counseling centers, registered doctors and therapists, medical care centers, memory clinics, nursing services, volunteer services and hospitals, with the aim of obtaining data that is as valid as possible. A special feature of the study is also that the three study regions have different geographical, demographic and socio-economic profiles and different population trends. While the rate of refusal for study participation could not be assessed due to time constraints in the recruiting institutions, there is little evidence towards a selection bias, as age (M = 61.8 years) and gender (72% women) of the final sample of informal caregivers in this study are quite similar to that of study samples in other studies on Bavarian informal caregivers (e.g. M_age_ = 61.3 years, 76% women [30]).

There are missing data in the follow-up. Of the 339 participating informal caregivers, 56% completed follow-up after a year. However, this is average for European studies involving informal caregivers [56]. Those dyads which dropped out of the study featured higher age, lower cognitive function, and more behavioral symptoms in the PWD as well as more comorbidities in the informal caregivers than non-dropouts. This is to be expected, as both severity of dementia and physical impairment of the caregiver may lead to study dropout due to institutionalization of the PWD, death of the participants, or the informal caregivers no longer feeling able to continue study participation. No effect could be observed of the dropped-out informal caregivers being more depressed at baseline than the non-dropouts. In addition, the interviews were conducted in the home environment of the PWD, which allowed a realistic picture of the living situation of the PWD to be obtained.

It was found that change in supervision time, a modifiable variable, predicted change in depressiveness. Due to the lack of experimental design however, a causal conclusion cannot be drawn without doubt regarding the relationship between these two variables. An interventional study aiming to impact the time spent on supervision would be required to do so. Nevertheless, longitudinal register research is still preferable over cross-sectional observation, and many different variables were able to be included in the analysis.

As suggested in the systematic review by Topp and colleagues [36], the depressiveness scores were multiplied by 4 in order to achieve a range of scores from 0 to 100 and to be better comparable with other studies using the WHO-5. However, it must be considered that this artificial transformation of the values feigns a higher accuracy than is actually present.

Finally, even though several studies found adequate sensitivity and specificity for the WHO-5 as a screening tool for depression [36], it should be stressed that a score below 50 does not prove the presence of clinical depression but should rather encourage to conduct further diagnostics. Thus, it cannot be said that 46% of the study population were clinically depressed but rather that clinical depression is likely in this group. However, the aim of the study was not to diagnose informal caregivers with depression but rather to find predictors of the change of depressive symptoms over time, which the study was able to achieve, as the WHO-5 is a valid instrument for screening for depression [36] and yields similar results as other depression scales [57–59].

### Practical implications

Following the results of this study, practical implications can be drawn in regard to both direct and indirect relief of informal caregivers of PWD. In order to possibly lower depressiveness of informal caregivers, supervision time of the PWD should be reduced. This can be done by promoting support services for informal caregivers so that they can take time for themselves to recuperate. Examples of such services might be day care centers, care services, organized neighborhood assistance as well as short-term or respite care. Support services are currently still used to a small extent [60–62]. The successor study to BayDem, digiDEM Bayern, plans to assess use of and need for support services in Bavaria as well as reasons why support services might not be used by caregivers and their PWD [63]. This way, potential barriers to service use might be found and actions for their removal might be recommended to major decision makers.

Indirect relief for the caregivers on the other hand can be achieved by trying to delay the course of the disease as much as possible to keep the necessary supervision time low. Some psychosocial interventions have proven successful in this aspect. One of them is the evidence-based, multi-component MAKS therapy, which includes exercises for social activation, sensorimotor activation, cognitive activation and activation of ADL. A randomized controlled study was able to show MAKS therapy’s effectiveness in stabilizing the outcomes “cognitive abilities”, “ADL abilities” and “neuropsychiatric symptoms” over 6 months as compared to usual care [64]. Thus, effective psychosocial interventions might be able to reduce the need for care by family caregivers by keeping practical everyday skills longer.

## Conclusions

Informal caregivers of PWD are at high risk of depression, leading to negative outcomes for those affected. A major factor influencing depressiveness longitudinally is the time spent supervising the PWD. Thus, support services which give the informal caregiver relief and time to themselves are of large importance to potentially decrease caregivers’ depressiveness.

## Data Availability

The datasets used and/or analysed during the current study are available from the corresponding author on reasonable request.

## Abbreviations

ADL: Activities of daily living
BSFC-s: Burden Scale for Family Caregivers (shortform)
iADL: instrumental activities of daily living
LSNS: Lubben Social Network Scale
MMSE: Mini Mental State Examination
NPI: Neuropsychiatric Inventory
PWD: Person/people with dementia
RUD: Resource Utilization in Dementia
WHO-5: WHO-Five Well-being Index
